# Templateless crystallization of holococcolith crystals visualized by intracellular site-specific three-dimensional microscopy

**DOI:** 10.1093/pnasnexus/pgag104

**Published:** 2026-04-07

**Authors:** Oz Ben-Joseph, Yu-Feng Meng, Lior Aram, Ikhlas Abu Freha, Zipora Lansky, Razi Safadi, Diede de Haan, Katya Rechav, Ifat Kaplan-Ashiri, Neta Varsano, Assaf Gal

**Affiliations:** Department of Plant and Environmental Sciences, Weizmann Institute of Science, Rehovot 7610001, Israel; Department of Plant and Environmental Sciences, Weizmann Institute of Science, Rehovot 7610001, Israel; Department of Plant and Environmental Sciences, Weizmann Institute of Science, Rehovot 7610001, Israel; Department of Plant and Environmental Sciences, Weizmann Institute of Science, Rehovot 7610001, Israel; Department of Plant and Environmental Sciences, Weizmann Institute of Science, Rehovot 7610001, Israel; Department of Plant and Environmental Sciences, Weizmann Institute of Science, Rehovot 7610001, Israel; Department of Plant and Environmental Sciences, Weizmann Institute of Science, Rehovot 7610001, Israel; Department of Chemical Research Support, Weizmann Institute of Science, Rehovot 7610001, Israel; Department of Chemical Research Support, Weizmann Institute of Science, Rehovot 7610001, Israel; Department of Chemical Research Support, Weizmann Institute of Science, Rehovot 7610001, Israel; Department of Plant and Environmental Sciences, Weizmann Institute of Science, Rehovot 7610001, Israel

**Keywords:** biomineralization, calcite, coccolith, cryoEM, nucleation

## Abstract

Biological crystallization generates some of the most intricate and diverse materials found in nature. Among the most striking examples are calcium carbonate structures called coccoliths, which are formed by unicellular marine algae. Despite the exquisite control over crystal orientations and arrangements, the mechanisms by which these cells control crystal nucleation remain poorly understood, largely due to the experimental difficulty of probing crystallization in vivo. Here, we report on intracellular crystallization during the formation of holococcoliths—superstructures assembled from rhombohedral calcite crystals. We establish a serial cryo-focused ion beam milling strategy to directly access the intracellular site where calcite precipitates. Cryo-electron tomography (cryoET) demonstrates a biomineralization mode where nucleation and growth of crystals occur within an isotropic environment and in the absence of any templating structures discernable by cryoET. Based on these observations, we propose a two-step mechanism, where an ordering step follows the initial random nucleation events. Such a process might be common to other biomineralization phenomena that evolve their order gradually.

Significance Statement Crystal nucleation has an inherent stochastic nature since it requires overcoming an energy barrier, making the control of timing and location of nucleation events challenging. Nevertheless, nature seems to evolve mechanisms to control nucleation, as many biologically formed minerals are characterized by precise polymorph, orientation, and timing of development. In this study, state-of-the-art cryo-electron microscopy is used to study the native-state organization at the site of crystal nucleation in holococcoliths. Evidently, the crystals nucleate in bulk solution, which is in contrast to the widely accepted dogma that organic templates regulate crystal nucleation. Such indirect ordering processes were discussed also in other biomineral systems and with the current methodologies might now be directly probed.

## Introduction

Organisms can regulate crystallization reactions that produce intricate, hierarchical minerals with remarkable precision and reproducibility (1–3). A crucial step in any controlled biomineral formation is nucleation, as it dictates the localization and orientation of each individual crystal in the final structure (4–6). It is generally accepted that nucleation in biological systems is controlled using organic matrices and surfaces as templates, where stereochemical interactions determine the site of nucleation (7, 8). These templates promote heterogenous nucleation via the reduction of the interfacial free energy of crystal nuclei and circumvent the stochastic nature of homogenous nucleation in the mineralization solution (9, 10). Many such organic templates were proposed in biological systems, and well-studied examples are the oriented carbonated apatite crystals of bones and the aragonite crystals of nacre. However, it is difficult to directly demonstrate the stereochemical relations involved, and other putative mechanisms are often discussed (11).

Hallmarks for biologically controlled nucleation are coccoliths, mineralized scales formed by a group of unicellular algae named coccolithophores (5, 12, 13). The most studied coccolith structures, heterococcoliths, are formed intracellularly, in a Golgi-derived vesicle named the coccolith vesicle (CV) (14–17). Heterococcoliths are composed of calcite crystals with highly convoluted morphologies, which are precisely arranged in radial arrays, where the crystallographic orientation of neighboring crystals alternates between two conserved directions. This necessitates a strict regulation of the nucleation stage, which is paradigmatically associated with the surface properties of an organic base plate scale, which is the first observed structure in the CV (5, 18).

Because heterococcolith crystals develop along the periphery of the oval base plate, initial suggestions postulated that the chemistry and topology of the base plate rim are conducive for oriented nucleation of calcite, which can be mediated via specific charged residues (19, 20). Nevertheless, this accepted view of direct stereochemical nucleation from the base plate surface is now challenged by new observations that point to other, indirect, mechanisms that might affect nucleation. For example, it was shown that a preceding dense phase forms via macromolecular recognition at the base-plate rim before crystal nucleation (12, 21). This suggests that mineralization is targeted to the correct location via interactions of the accompanying macromolecules. In addition, nanoscale imaging revealed that some of the alternating crystal unit types are not in direct contact with the base plate in both initial and mature structures, which complicates the notion that epitaxy can be solely responsible for nucleation control (22). Altogether, the regulation of coccolith crystal nucleation is still an unsolved enigma.

During their life cycle, coccolithophores produce an additional type of mineralized scales, called holococcoliths (23, 24). These coccoliths are composed solely of calcite crystals with rhombohedral morphology, similar to the most stable morphology of calcite. Structural diversity among holococcoliths produced by different species arises from the varying ways in which hundreds of these rhombohedral crystals are packed into intricate three-dimensional (3D) assemblages (25). Compared with heterococcoliths, holococcolith formation is understudied, with only recent studies showing that holococcoliths are formed intracellularly inside a CV that is more voluminous than that of heterococcoliths (26–28). Intriguingly, base plates with similar properties are found within the CV of the two distinct coccolith types (29).

In holococcoliths, as opposed to heterococcoliths, only a small subset of crystals in the mature structure directly contacts the organic base plate. This arrangement raises the possibility that the base plate does not play a direct role in templating the positions of crystals (15, 27). Nevertheless, the holococcolith structure can be as regulated and ordered as the heterococcolith architecture, raising the question of what might be the structural entity that plays a role in crystal arrangement. Since traditional transmission electron microscopy (TEM) sample preparation of biological specimens includes dehydration, slow fixation, and contrast generation by heavy metal staining, it is plausible that undetected cellular features in the CV regulate or template the nucleation of the crystals and were not documented to date.

The preservation limitations of conventional biological TEM can be addressed by cryogenic methods involving the rapid vitrification of cells and imaging them at a cryo-hydrated state, known as cryo-electron tomography (cryoET) (30, 31). This approach provides exceptional preservation, allowing for the study of the native conformation of cellular structures and dynamic chemical phases in situ, and with nanoscale resolution (32–34). Recently, such cryoET studies demonstrated the effects of membrane confinement on crystal morphogenesis in heterococcoliths (22, 35). In the context of holococcolith formation, where critical stages of nucleation and structural assembly remain unresolved, cryoET has the potential to uncover previously undetected factors and resolve these longstanding questions.

In this study, to assess the structural determinants involved in the formation of holococcoliths, we analyze the intracellular environment of crystal nucleation and growth using cryoET. This approach enables us to characterize the environment of growing crystals with native contrast at high resolution and sensitivity. The in situ nanoscale imaging shows that crystals nucleate without any structural relation to a detectable template and are reorganized to ordered superstructures after formation.

## Results

**Figure 1:**
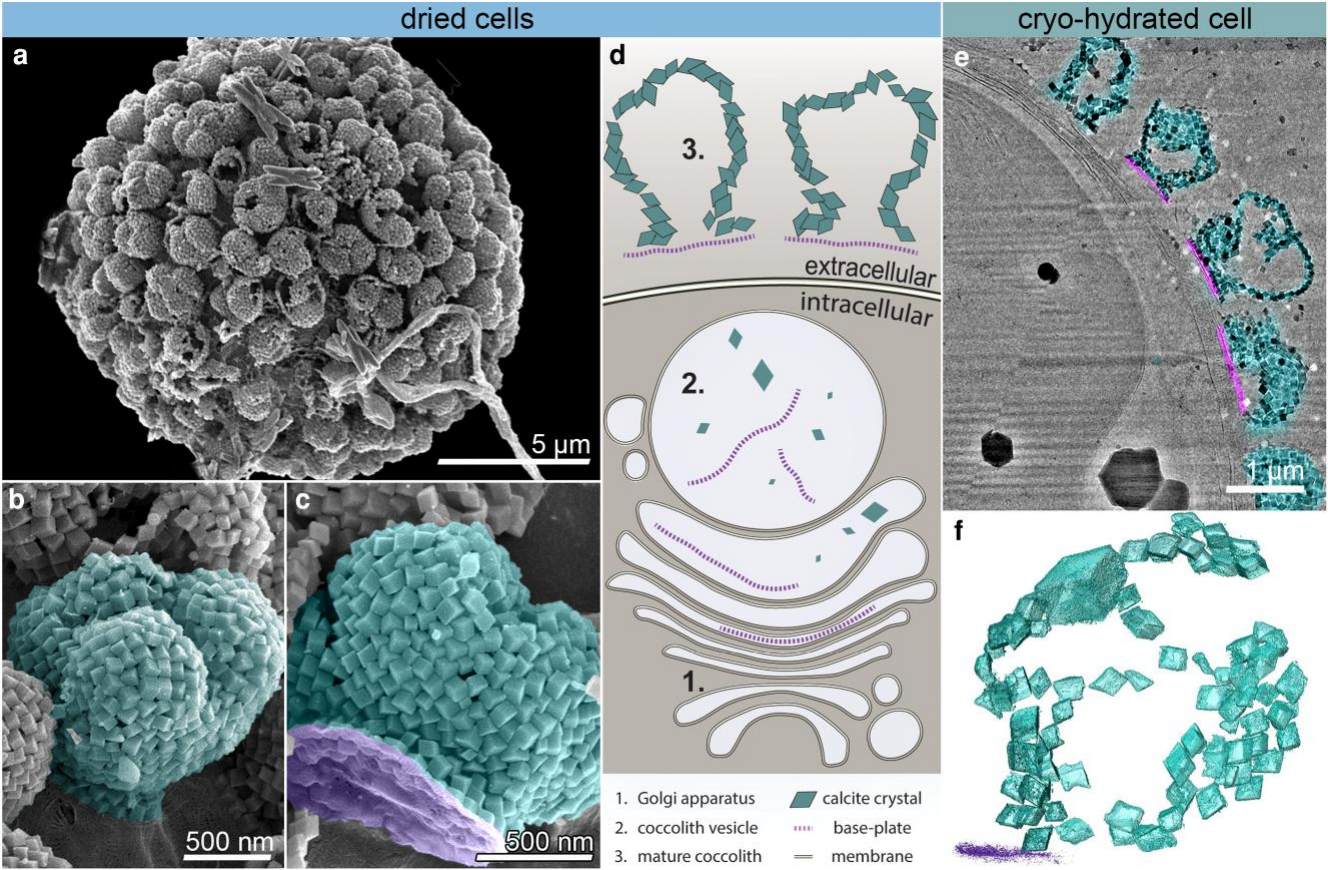
The cellular organization and holococcolith structure of *Calyptrosphaera* sp. a) SEM micrograph of a critical point–dried cell showing the extracellular holococcolith cover. b, c) False-colored SEM micrographs of individual coccoliths (cyan) and the associated organic base-plate scale (magenta). d) Schematic of the coccolith formation pathway based on previous studies: (1) organic scales are first formed in Golgi-derived vesicles; (2) crystals nucleate and grow within a spacious intracellular CV; and (3) the mature holococcoliths cover the cell surface. e) CryoTEM micrograph of a lamella milled through a plunge frozen cell, showing intracellular compartments and the extracellular holococcolith layer. Crystals (cyan) and base plates (magenta) are highlighted. f) 3D Surface rendering of a cryoET dataset showing a 200-nm section within a mature extracellular holococcolith.

We investigated holococcolith formation in *Calyptrosphaera* sp. cells. The holococcoliths produced by this species consist of hundreds of uniformly sized calcite crystals, each ∼100 nm across, tightly packed into a balloon-like structure (Figure 1a and b). In addition to crystals, the mature holococcolith includes an organic base plate (Figure 1c). However, unlike heterococcolith structures, only the basal layer of crystals is in direct contact with this base plate (Figure 1c). Previous studies have shown that holococcolith formation occurs within a spherical CV, ∼1 µm in radius, where crystals develop in the presence of the base-plate scale (schematic in Figure 1d).

To visualize intracellular crystal formation at the single-cell level using cryoET, a substantial portion of the algal culture should be actively forming holococcoliths. To achieve this, we induced calcification by applying an acid treatment that dissolves existing coccoliths, a procedure used in previous studies (35, 36). Culture aliquots containing actively calcifying cells were rapidly vitrified without chemical fixation or staining, and thinned to a 200-nm electron-transparent lamella using a focused ion beam at cryo conditions (cryoFIB), within a dual-beam FIB–scanning electron microscope (SEM). The vitrified lamellae are then transferred to a 300-kV cryoTEM microscope for imaging, where high-resolution images are collected for various regions of interest such as the extracellular coccoliths outside of the cell (Figure 1e). A series of 2D projection images from a region in the lamella are acquired at varying tilt angles, which are computationally reconstructed into a 3D dataset. 3D surface rendering of the crystals enables to visualize the holococcolith fraction that resides in the lamella (Figure 1f).

**Figure 2:**
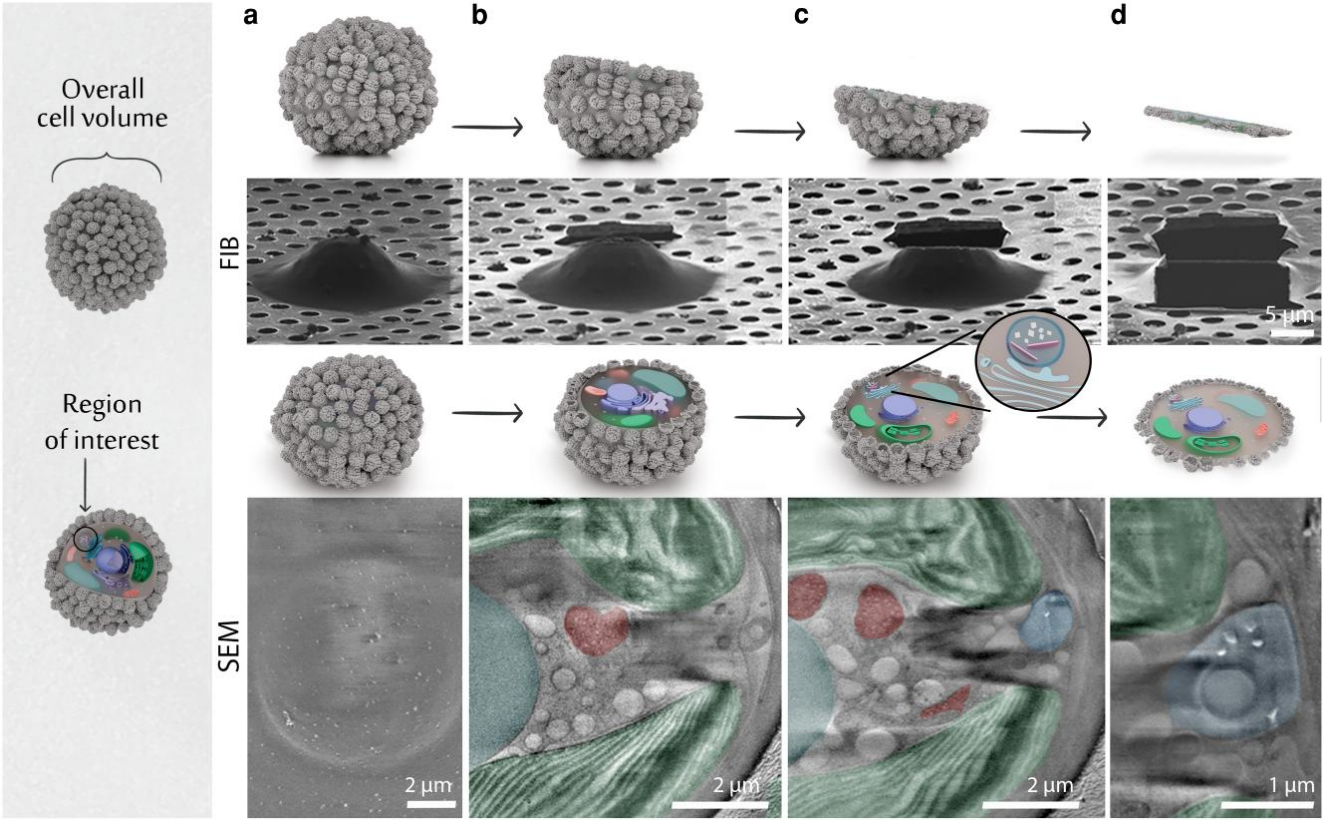
Site-specific lamella preparation for CV data collection. a–d) Overview of FIB milling and SEM imaging steps. a) Vitrified whole cell mounted on a TEM grid. b) Top cellular layers are progressively removed by 50 nm steps of FIB milling, with intermitted SEM imaging of the exposed intracellular surface. c) The CV (highlighted in blue) is identified based on the presence of high-contrast crystals. d) Final ∼200 nm thick lamella is prepared by thinning the remaining cellular material beneath the exposed surface. In the SEM images chloroplasts are highlighted in green, mitochondria in red, and the nucleus in cyan.

A major limitation of in-cell cryoET is the low yield of lamella preparation, which originates from the complicated milling procedure and the fragility of the lamellae. Lamella preparation is usually performed at the center of the cell with the expectation that major cellular components will be represented in the imaged volume. However, the holococcolith CV is a small organelle, occupying <1% of the cell's volume (radius of 1–1.5 µm for the vesicle and 10 µm for the cell). This means that using such untargeted milling will result in a prohibitively low probability of finding a CV within the lamella volume. To mitigate this challenge, we developed a serial slice-and-view workflow that enables site-specific lamella milling (Figure 2).

This approach relies on the ability to combine stepwise milling intervals, with high-resolution cryoSEM imaging of the exposed cell surface at each step. First, an intact cell, covered by a layer of vitrified seawater and situated at the central area of a TEM grid square, is identified using SEM and FIB imaging (Figure 2a). The top of the cell is then milled and viewed by serial FIB milling and SEM imaging (Figure 2b). The cryoSEM imaging mode is used during the serial FIB milling progress to locate the slice where a CV is exposed. CV detection is based on its unique structural features, namely high-contrast crystals within a vesicle (Figure 2c). Upon CV detection, a lamella is produced by milling the remaining cellular material beneath the exposed cell surface (Figure 2d). Using this method, we generated lamellae containing CVs from ∼15 cells (Figure S1).

**Figure 3:**
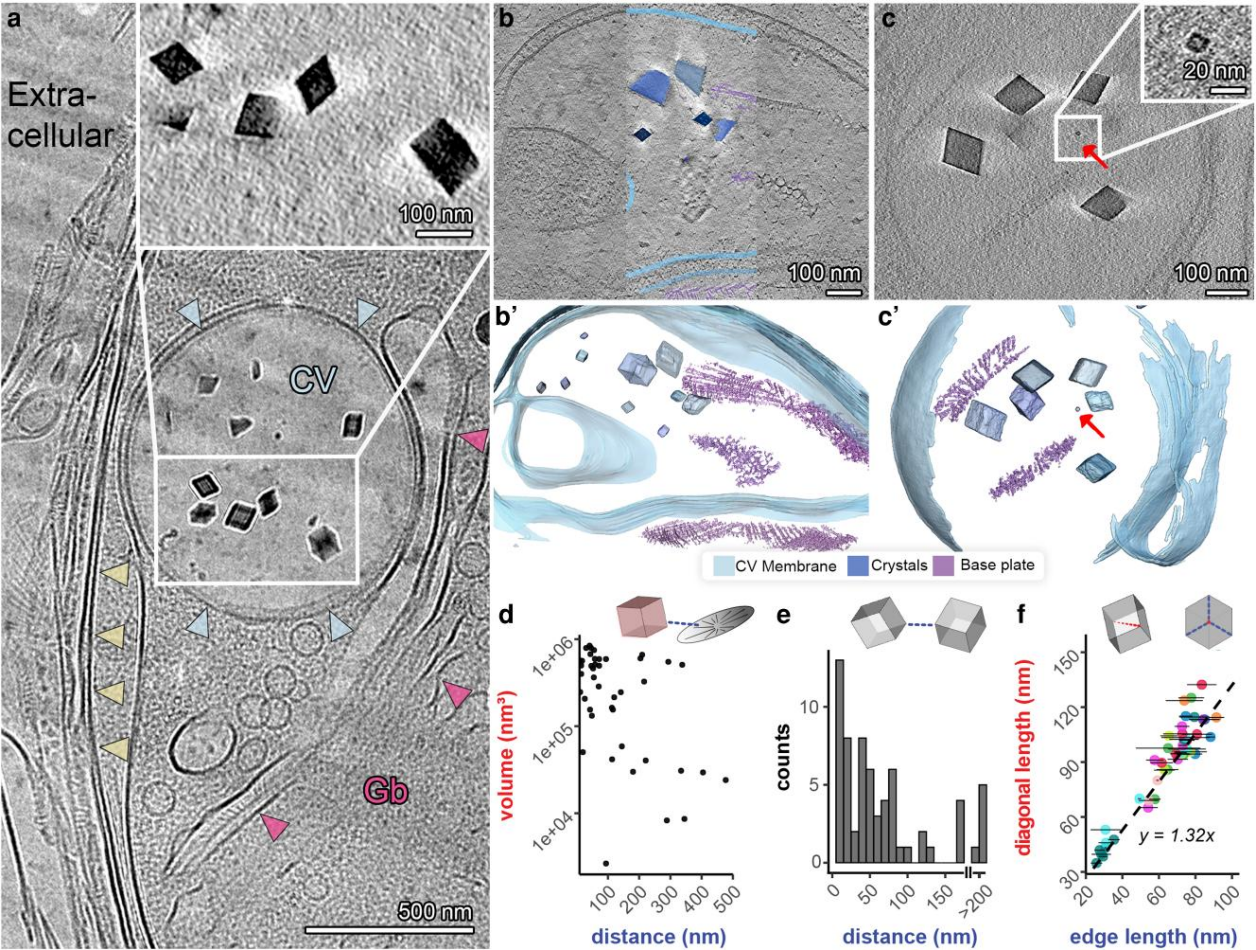
Structural properties of the CV revealed using cryoET. a) CryoTEM projection image of a lamella showing the CV (blue arrowheads), Golgi apparatus (pink arrowheads), cytoplasm, plasma membrane (yellow arrowheads), and extracellular layers. Inset: A slice from the reconstructed tomogram containing the CV's crystals. b, c) Tomographic slices, and (b′, c′) the corresponding surface rendering segmentations, of CVs containing crystals of varying sizes alongside several scales. The smallest observed crystal of ∼10 nm is indicated by a red arrow in c and c′. d) Crystal to nearest scale distance plotted as a function of crystal volume. No relation is observed between small crystals and short distances. e) Distribution of nearest-neighbor distances among CV crystals. f) Morphological analysis of CV crystals, showing the ratio between the diagonal and edge lengths. The expected trend for a perfect rhombohedron (dashed line) is included for comparison. Panels (d–f) are based on measurements from surface-rendered tomographic data.

The high resolution, sensitivity, and superior structural preservation offered by cryoET enable direct visualization of the nanoscale environment in which holococcolith crystals form. In these datasets, the CV appears spherical and spacious, hosting several randomly scattered crystals within its lumen (Figure 3a), in accordance with previous room-temperature microscopy works. The ∼200-nm-thick CV sections usually include several crystals with a size range of 10 to 100 nm, alongside several organic scales (Figure 3b and c). The crystals are scattered within the vesicle in seemingly random locations, with no specific relation to the organic scales or membranes (Figure 3b’ and c’).

The resolution of these datasets is sufficient to distinguish macromolecular complexes such as the two lipid leaflets of the CV membrane (Figure 3b) and cytoskeleton elements such as actin filaments and microtubules, and yet, most of the observed crystals are not in contact with any discernible structure. Rather, the immediate surrounding of both crystals and organic scales consists of the same homogeneous lumen. An interesting observation is that more than one organic scale can be observed in most CVs. The cells produce both mineralized base plates and unmineralized organic scales, which we cannot differentiate based on structural aspects. We quantitatively analyzed all the collected datasets to determine the interactions between crystals and the organic scales within the CV. First, we measured the minimum distance between each crystal and the closest organic scale. This analysis shows that most crystals are found at distances of >50 nm from a scale, with no correlation between crystal volume and its proximity to a scale (Figure 3d). In other words, in contrast to heterococcolith crystals, there is no tendency of the smaller crystals to be closer to a scale. These geometrical observations make templated nucleation from the base plate an unlikely scenario.

Another putative scenario is that crystal nucleation occurs on the surface of neighboring crystals. This may seem likely since extracellular holococcolith crystals are densely packed, with a single crystal often contacting three other crystals (Figure 1f). However, measuring crystal-to-crystal distances of nearest neighbors within the CV shows a wide distribution of distances where most crystals are >10 nm apart (Figure 3e). This dispersed crystal arrangement within the CV, without close crystal-to-crystal contacts, further supports the notion of untemplated crystal nucleation in the CV.

To evaluate the crystallographic habit of growing holococcolith crystals, we analyzed the morphology of crystals at various sizes within the CV. As opposed to the common trait of heterococcolith crystals, which interlock with neighboring crystals, leading to a deformed crystallographic habit, all holococcolith crystals that were not cut by the FIB showed a clear rhombohedral habit with no indications of intergrowth of adjacent crystals. The diagonal length along the *c*-axis of the rhombohedron and the edge lengths of the rhombohedron were measured. Plotting the ratio of the rhombohedron diagonal length to its edge length shows a size-dependent trend that closely resembles an ideal rhombohedron (Figure 3f). This analysis indicates that the crystals acquire and maintain their rhombohedral morphology from early to late growth stages. Altogether, the analyses of the intracellular crystals point to untemplated nucleation of calcite rhombohedra within the CV.

**Figure 4:**
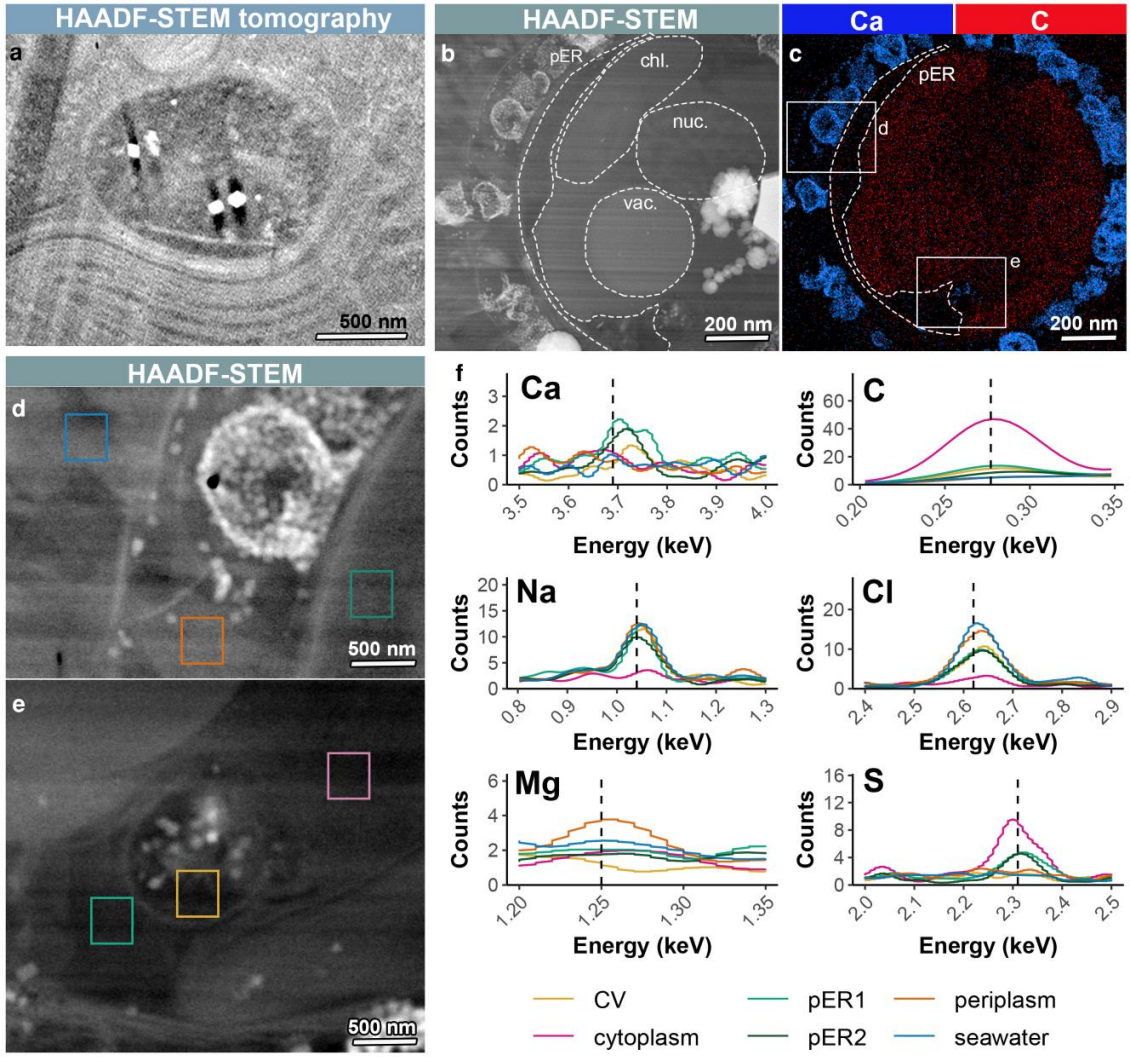
Mapping elemental compositions of cellular compartments using cryoSTEM-EDS. a) Tomographic slice from a HAADF-STEM dataset, highlighting the dark contrast of the CV lumen compared with the cytoplasm and calcite crystals (brighter pixels indicate higher electron density). b) HAADF-STEM image of a lamella, showing intracellular organelles, including the nucleus, vacuole, chloroplast, CV, and pER, as well as extracellular regions such as the periplasm and surrounding seawater medium. c) EDS elemental maps of calcium (Ca) and carbon (C) from the lamella shown in (b). The pER appears as a region with a low carbon signal. d–e) Higher magnification views of the CV (d), and periplasm (e), regions from the lamella in (b) and (c). Colored rectangles indicate areas where EDS spectra are presented. f) Color-coded EDS spectra of selected elements corresponding to the regions marked in (d) and (e).

The cryoET data show that a homogeneous lumen is surrounding the crystals and scales in the CV. The lumen appears with a clear texture, lacking the granular composition observed in the adjacent cytoplasmic solution, indicating a low macromolecular content in the CV (Figure 3a). We examined the CV lumen using high-angle annular dark-field (HAADF) tomography in scanning transmission electron microscope (STEM) mode, which uses elastically scattered electrons for contrast generation, and serves as an indication of electron densities at different locations on the sample. HAADF-STEM tomography shows that the CV lumen is darker than the neighboring cytoplasm, indicating a lower electron density (Figure 4a). It is probable that this originates from a low concentration of macromolecules.

We analyzed the elemental composition of the CV lumen using cryoSTEM energy dispersive X-ray spectroscopy (EDS). Detecting EDS signals from biological solutions is not a trivial task, given the low concentrations of low-Z elements. To increase the signal intensities, we prepared lamellae with an increased thickness of 300 nm and used a highly sensitive EDS detector in a quadrupole configuration. Using cryoEDS mapping in correlation to cryoSTEM imaging, the extracellular coccoliths stand out for their Ca signal and the cytoplasm for the C content (Figure 4b and c). Comparing spectra collected from specific regions shows that the vitrified seawater around the cells shows only Na and Cl peaks, their most abundant elements, with a faint Mg signal (Figure 4d–f). In the cytoplasm, only S and C peaks are detected, likely originating from macromolecules. Ca was not detected in both seawater and CV, placing the detection limit above the 10 mM concentration of Ca in seawater, similar to other cryoEM techniques (37).

Interestingly, the only detectable Ca peak was observed within a large intracellular organelle (Figure 4b). This organelle, which displays low carbon content (Figure 4c) and a homogeneous, electron-lucent appearance under bright-field TEM (Figure S2) that is atypical of cytoplasm, is identified here as the peripheral endoplasmic reticulum (pER). The pER forms extensive contact with both the plasma membrane and the CV (Figure 4b and c), and contains not only Ca but also Na and Cl at concentrations comparable to seawater (∼0.5 M, Figure 4f). Notably, the lumen of the CV exhibits similar Na and Cl levels (Figure 4f). These observations may suggest the existence of a seawater–pER–CV transport route, wherein Ca is concentrated within the pER before being delivered to the CV, where crystallization takes place.

**Figure 5:**
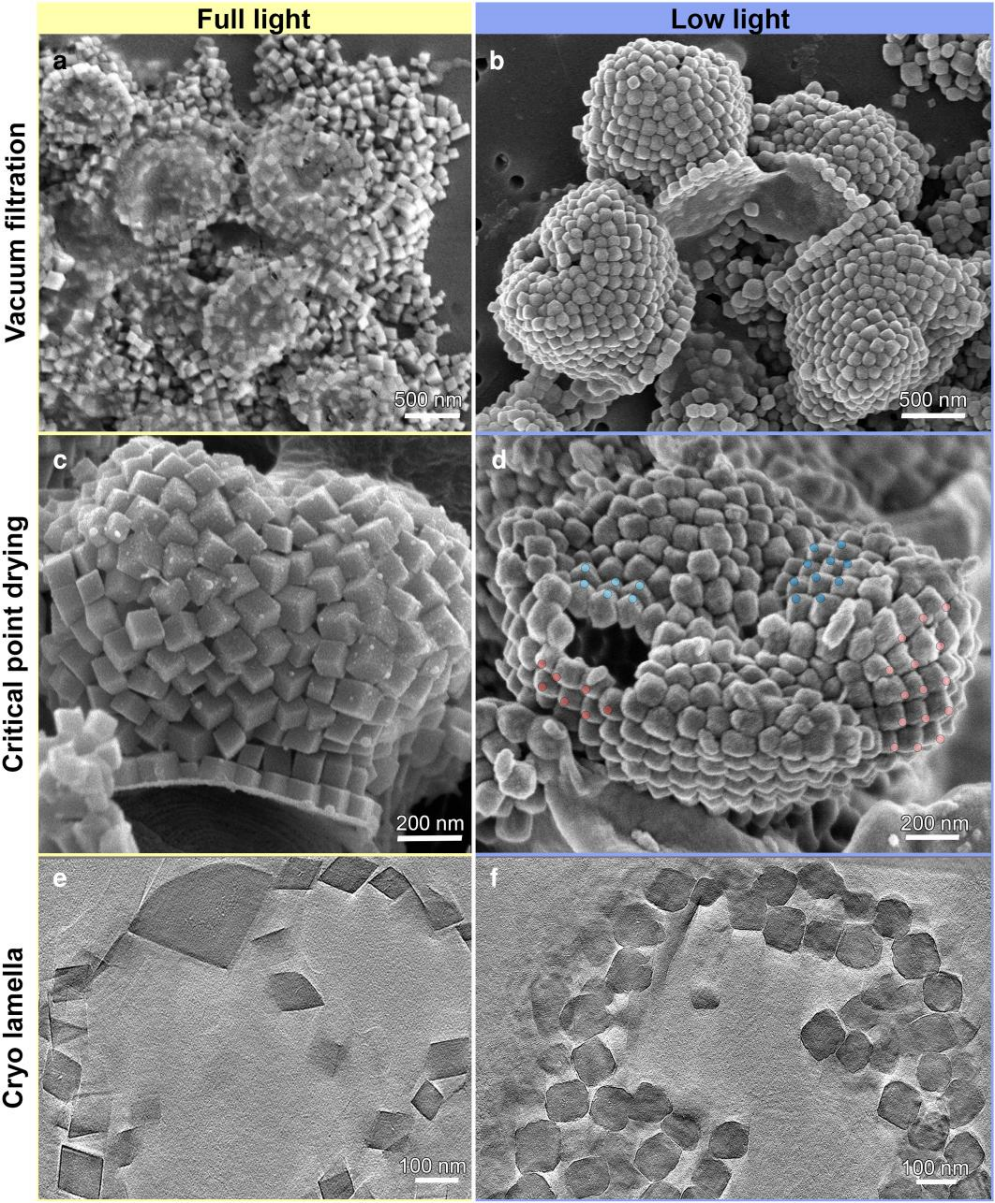
Effect of growth conditions on holococcolith morphology. a) SEM image of holococcoliths from full-light cultures that collapse into flattened aggregates after dehydration by vacuum filtration. b) Holococcoliths from low-light cultures retain their intact 3D structure. c, d) Critical point–dried holococcoliths show that full-light cultures maintain their disordered ultrastructure but lack consistent crystal orientation (c), while low-light cultures exhibit patches of co-oriented crystals, indicated by colored circles (d). e, f) CryoET reconstructions highlight differences in crystal morphology: full-light crystals display sharp rhombohedral facets (e), whereas low-light crystals show rounded edges and rough surfaces (f).

The disordered organization of the holococcolith crystals in the cultured *Calyptrosphaera* sp. cells contrasts with the intricate arrangements of holococcolith samples collected from natural environments. Since our structural data shows that coccolith architecture is decoupled from crystal nucleation and growth, we wanted to explore whether environmental conditions can affect holococcolith properties. To test the role of growth rate, we reduced light intensity to sustain only minimal growth that was maintained through infrequent culture dilutions (doubling of cell numbers was in the order of months). Under these conditions, holococcoliths of *Calyptrosphaera* sp. displayed striking differences in morphology and preservation. Whereas conventional SEM preparation caused collapse of holococcoliths from normal-light cultures into flattened aggregates, low-light cultured holococcoliths retained their intact 3D structure (Figure 5a and b).

Critical-point drying further revealed that growth conditions strongly influence crystal organization. In normal-light cultures, holococcoliths maintained intricate ultrastructure but lacked consistent crystal orientation (Figure 5c), as also observed in cryoET reconstructions (Figure 1f). In contrast, holococcoliths from low-light cultures exhibited patches of co-oriented crystals, suggesting cellular control over orientation during assembly (Figure 5d). Moreover, while crystals from full-light conditions showed sharp rhombohedral morphology by cryoET, those from low-light cultures were more rounded and irregular (Figure 5e and f). Together, these observations demonstrate that growth conditions modulate both the structural integrity of holococcoliths and the degree of crystallographic control.

## Discussion

The dynamics of biologically regulated crystallization remain a challenge to observe and understand. In this study, we developed a workflow enabling native-state in situ observations of crystal nucleation and growth during holococcolith formation. Our observations suggest that holococcolith crystals nucleate without any templating or ordering that can be visualized with cryoET. Untemplated nucleation is deduced from the absence of structures in the immediate surroundings of crystals and the lack of spatial correlation between early-stage crystals and the surface of base plates. Such biomineralization strategy is unusual among most of the familiar pathways where crystals nucleate and grow at their final location in the biomineral structure. Our data cannot rule out heterogenous nucleation from small enough moieties that are not detectable. Nevertheless, it is consistent with a less expected scenario where homogenous nucleation is the first step and crystals then relocate and rearrange. If this is the case, it will be interesting to explore possible similarities with oriented attachment pathways.

One of the most intriguing aspects of holococcolith biomineralization is the assembly process of the rhombohedral calcite crystals into the complex and species-specific superstructures. After the initial step of untemplated nucleation, the crystals are growing in the CV, keeping their rhombohedral shape. This is in contrast to the intergrowth of heterococcoliths that gives rise to very convoluted and interwoven crystal morphologies. The presence of the various crystal sizes within the same CV raises questions about how size homogeneity is maintained. Since crystals at all growth stages are simultaneously present in the CV and experience the same solution environment, a shift in solution conditions cannot be accountable for the cessation of growth. However, growth termination at a specific size could be driven by a growth barrier resulting from cumulative effects, such as impurity incorporation causing lattice strains and creating a barrier to further growth.

The rhombohedral morphology provides further support to the notion of an initial growth step that is followed by an ordering step. This is because in heterococcoliths, where growth is coupled to the final order, the crystals intergrow to deformed rhombohedral habits. The more ordered coccoliths that formed under low-light conditions are pointing to a similar mechanism. We suggest that the ordering process is dependent on the time that it takes to form the coccolith. When metabolism is slow due to energy availability and coccolith formation is accordingly slowed down, the crystals have more time to explore orientational rearrangements and to achieve compact and ordered packing. Since we could not directly detect the step of ultrastructure assembly intracellularly, it is possible that some ordering continues after exocytosis. However, a recent study on *Calcidiscus leptoporus* was able to detect mature holococcoliths within the cell (28). Future work on more holococcolith-forming species using approaches such as live-cell imaging techniques may reveal more information about this enigmatic process.

The HAADF-STEM tomography data, together with the cryoEDS mapping, allow to add compositional information to the structural information. It is surprising that the CV lumen contains a low electron density solution, as the saturation with respect to calcium carbonate should be higher compared with the cytoplasm. Therefore, it can be deduced that macromolecular content dominates the different contrast between the crystallization solution and other cellular environments, and via regulation of their presence and concentration the cell controls the kinetics and other characteristics of crystallization. In addition, the spatial interaction with the CV and the Ca content of the pER suggests that Ca is transported from seawater to the CV via the pER. Within the CV, Na and Cl are present at similar levels as in the pER and seawater. It is important to note that EDS of intracellular environments is very challenging, both from the perspective of sample preparation and due to beam damage, making this initial successful implementation a promising avenue for future studies.

## Conclusion

Holococcolith structures are formed through a highly regulated cellular process, resulting in monodispersed calcite rhombohedra assembled into intricate arrangements. Our observations indicate that holococcolith crystals nucleate in the absence of detectable templating surfaces, and growth occurs in a simple, low density, and unconfined solution. This crystallization mode facilitates a uniform population of crystals but necessitates an additional assembly step for structural organization. Future research should focus on elucidating the mechanisms by which cells orchestrate crystal size uniformity and crystal positioning and assembly postcrystallization.

## Supplementary Material

pgag104_Supplementary_Data

## Data Availability

The data underlying this article are available in the article and in its online Supplementary material.
