# Supplementary material for: Templateless crystallization of holococcolith crystals visualized by intracellular site-specific three-dimensional microscopy
Source: PNAS Nexus. 2026 Apr 7;5(4):pgag104. doi: 10.1093/pnasnexus/pgag104 (PMC13089448; doi:10.1093/pnasnexus/pgag104)
Supplement: pgag104_Supplementary_Data [file pgag104_supplementary_data.pdf]

Supplementary Information

Template-less crystallization of holococcolith crystals visualized by intracellular site-specific 3D microscopy

Oz Ben-Joseph, Yufeng Meng, Lior Aram, Ikhlas Abu Freha, Zipora Lansky, Razi Safadi, Diede de Haan, Katya Rechav, Ifat Kaplan-Ashiri, Neta Varsano, and Assaf Gal

Methods

Cell Cultures

Clonal cultures of *Calyptrosphaera sp.* (RCC1181) obtained from the Roscoff Culture Collection (www.roscoff-culture-collection.org) were grown in sterile-filtered seawater collected from the Mediterranean Sea. Culturing was conducted in a temperature-controlled culture room at 18 °C with a 16h:8h light:dark cycle.

Low light culture

To slow down culture growth, we covered the bottom 5 cm of the culture flasks with a thick black paper. This allowed the entrance of light only from the top of the flask. The light intensity was less than 5 % in comparison to normal light conditions. At these reduced light conditions growth rate was too slow to quantitatively determine growth rates. Culture maintenance procedure was the following: initial inoculum consisted of 10^5 cell*mL^{-1} , cell numbers were counted every two weeks, and if they surpass 1.2*10^5 cell*mL^{-1} the culture was diluted back to 10^5 cell*mL^{-1} . On average, doubling time at these conditions is in the order of 2-4 months.

SEM sample preparation

To minimize collapse of structures caused by water surface tension, specimens were dried by critical point drying (CPD). Cultures were subjected to a series of graded ethanol/water washes

(30%, 70%, and finally 100%). They were then dried at the CO_2 critical point using a BAL-TEC CPD 030 system fitted with a fine-particle holder suitable for material between 2 and 30 µm (Tousimis Research Corporation). After drying, the samples were placed onto aluminum stubs (EMS 75230) and coated with a ~2.5 nm iridium layer via sputtering. Imaging was performed with a Zeiss Ultra 55 scanning electron microscope at 2 kV, employing an InLens detector.

Calcification induction

To maximize the chance of observing intracellular stages of holococcolith calcification by cryoET, we enriched the proportion of calcifying cells in the culture by acid decalcification treatment that was described previously (28). Cultures in exponential stage (~10^5 cells mL^{-1}, 50 mL total volume) were subjected to an acid decalcification step. The medium pH was first lowered to 5 by the gradual addition of 1 M HCl, after which 1 M NaOH was added to readjust the pH to 8.2. Throughout the treatment, which took about 5 minutes, cultures were continuously mixed with a magnetic stirrer, and pH changes were monitored using a pH electrode. Following neutralization, cells were incubated for 12 h a recovery period that coincides with the peaking of calcification rates.

Plunge freezing

Acid decalcified *Calyptrosphaera sp.* cells at 12 hr post treatment were cryo-fixed, without any washing or staining step, by plunge-freezing on carbon coated copper grids (Quantifoil Micro Tools). 5 µl of cell suspension at a density of ~2×10^5 cells/ml was pipetted on the carbon coated side and blotted for 1 s from the back side. Grids were then immediately plunged into a liquid ethane bath cooled by liquid nitrogen.

Site- Specific Lamella Milling

Vitrified grids were loaded into pre-cooled FIB-SEM microscope (Zeiss Crossbeam 550). Directed FIB lamella milling was carried out by stepwise FIB milling and SEM imaging of a single cell, in a similar manner to the process of slice and view tomography described above. The thickness of the steps varied from ~500 nm where no parts of the Golgi were detected to 50 nm thick steps where the Golgi was evident. Once the CV is identified, additional milling steps underneath the exposed surface were conducted, leaving a 200 nm thick lamella for electron tomography, and 500 nm thick for STEM- EDS experiments.

Cryo Electron Tomography

Cryo-electron tomography data of coccolith forming vesicles were collected from 15 cells. The tilt series were acquired using a Titan Krios G3i TEM (Thermo Fisher Scientific, Waltham, MA, USA), operating at 300 kV. Tilt series were recorded on a K3 direct detector (Gatan Inc., Pleasanton, CA, USA) installed behind a BioQuantum energy filter (Gatan Inc., Pleasanton, CA, USA), using a slit of 20 eV. All tilt series were recorded in counting mode at a nominal magnification of 33,000 ×, corresponding to a physical pixel size of 0.26 nm, using the dose-symmetric scheme starting from the lamella pre-tilt of −16° or 16° (dependent on grid orientation) and with 2° increments to acquire ~ 60 tilts for each tomogram. Tilt series were taken at a defocus range of 3–7 μm, using an objective aperture of 100 μm inserted. Tilt series were acquired using an automated low-dose procedure with a total dose of 100 to 120 e-/Å^2. Tomographic reconstructions were done using IMOD software using patch tracking for alignment.

Segmentation of tomographic datasets

Tomographic data were segmented using Amira software (v2021.2, Thermo Fisher Scientific, Waltham, MA, USA). Individual crystals were segmented manually based on contrast thresholding. Volume measurements were extracted using the "Label Analysis" module. Length and distance analyses were performed manually within the Amira environment. The orientation of the calcite C-axes was determined based on crystal morphology, with azimuthal (Θ) and polar (Φ) angles calculated relative to the dataset's XY plane.

Cryo EDS

Site directed lamellae were milled to a thickness of 200 and 500 nm with a 12° angle to the grid plane in a similar manner to cryo-ET lamella, with the exception of using unclipped grids. Unclipped grids were transferred into Thermo Fisher Scientific Talos F200X G2 equipped with high-speed energy dispersive X-ray spectroscopy system using a Gatan 914 cryo-holder. The microscope was operated at an accelerating voltage of 200 kV, with the gun lens set to 4.4, spot size 6, and 150 μm condenser aperture to optimize beam current and spatial resolution. Each lamella was imaged in HAADF mode. Then EDS was acquired from regions of interest for 3.5 minutes with a resolution of 1024x1024 pixels and a dwell time of 200 ns per pixel. Data acquisition and quantification were conducted using Thermo Scientific's Velox software.

Supporting figures

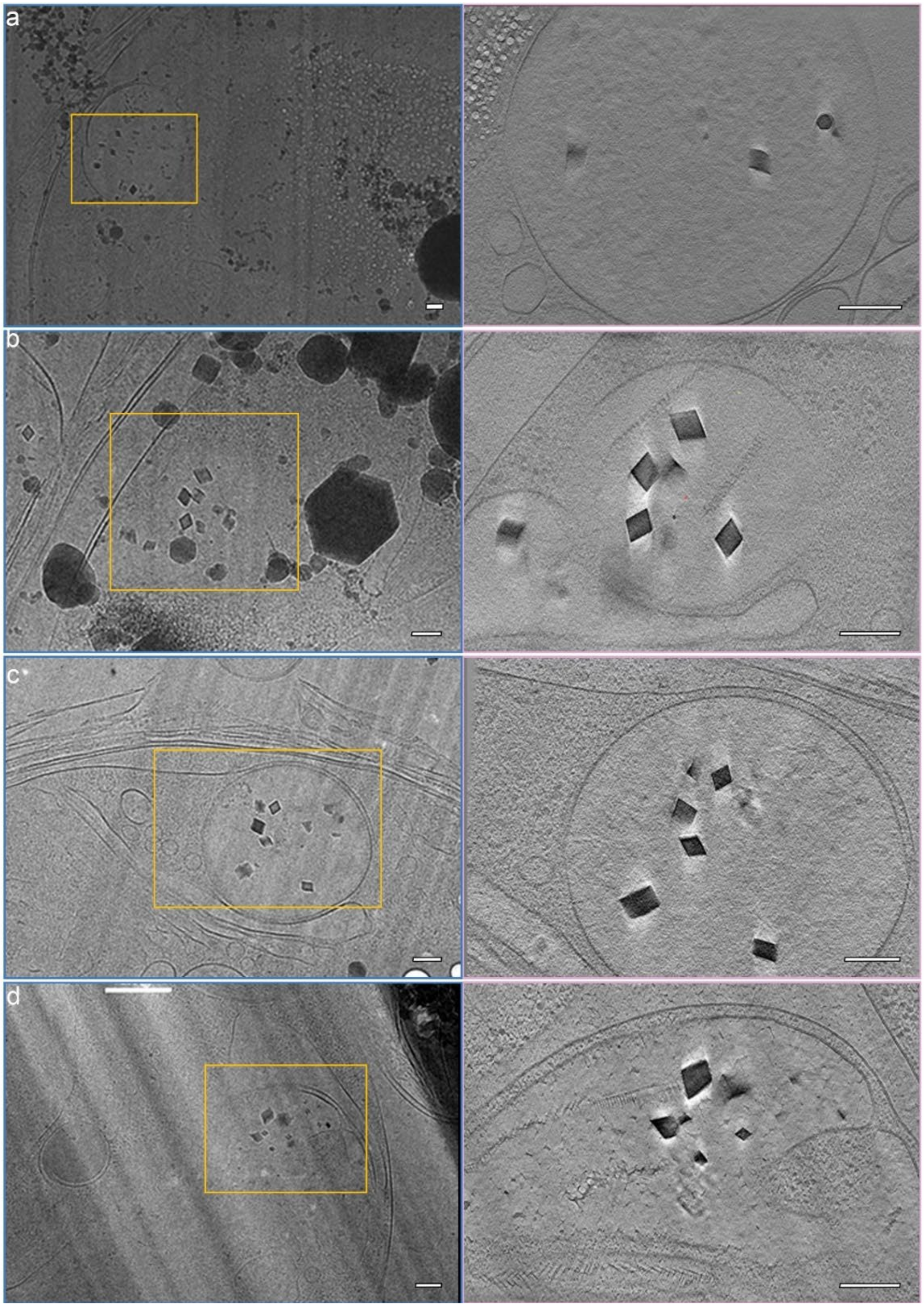

Figure S1 panel 1/4. The 15 cryoET datasets collected for this study (a-o) and the one cryoSTEM tomography dataset (p). For each dataset the left panel shows a cryoTEM image of the lamella, with one or two areas selected for tomography marked by rectangles. The right panels show representative slices in the cryoET data. Scale bars are 200 nm.

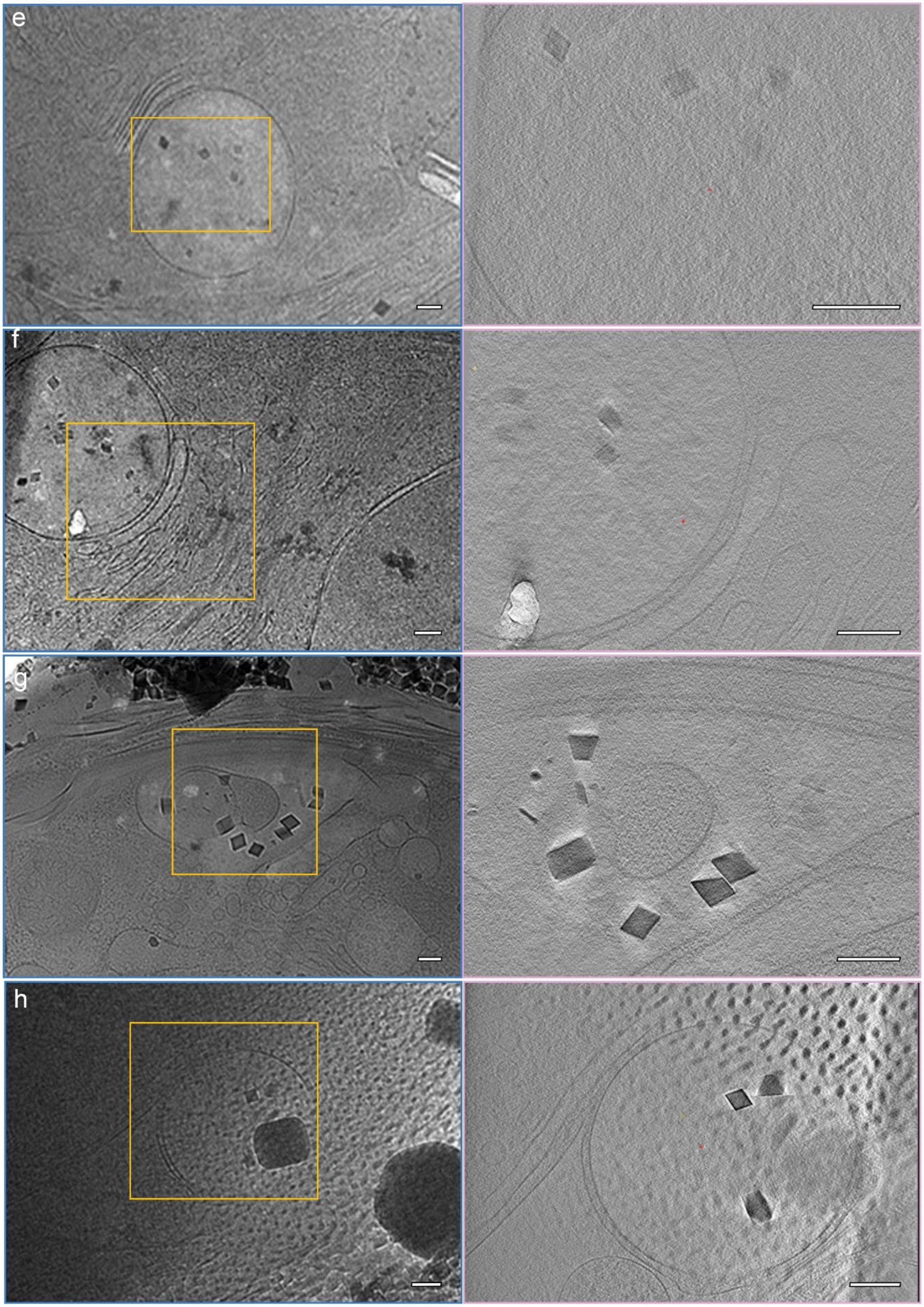

Figure S1 panel 2/4.

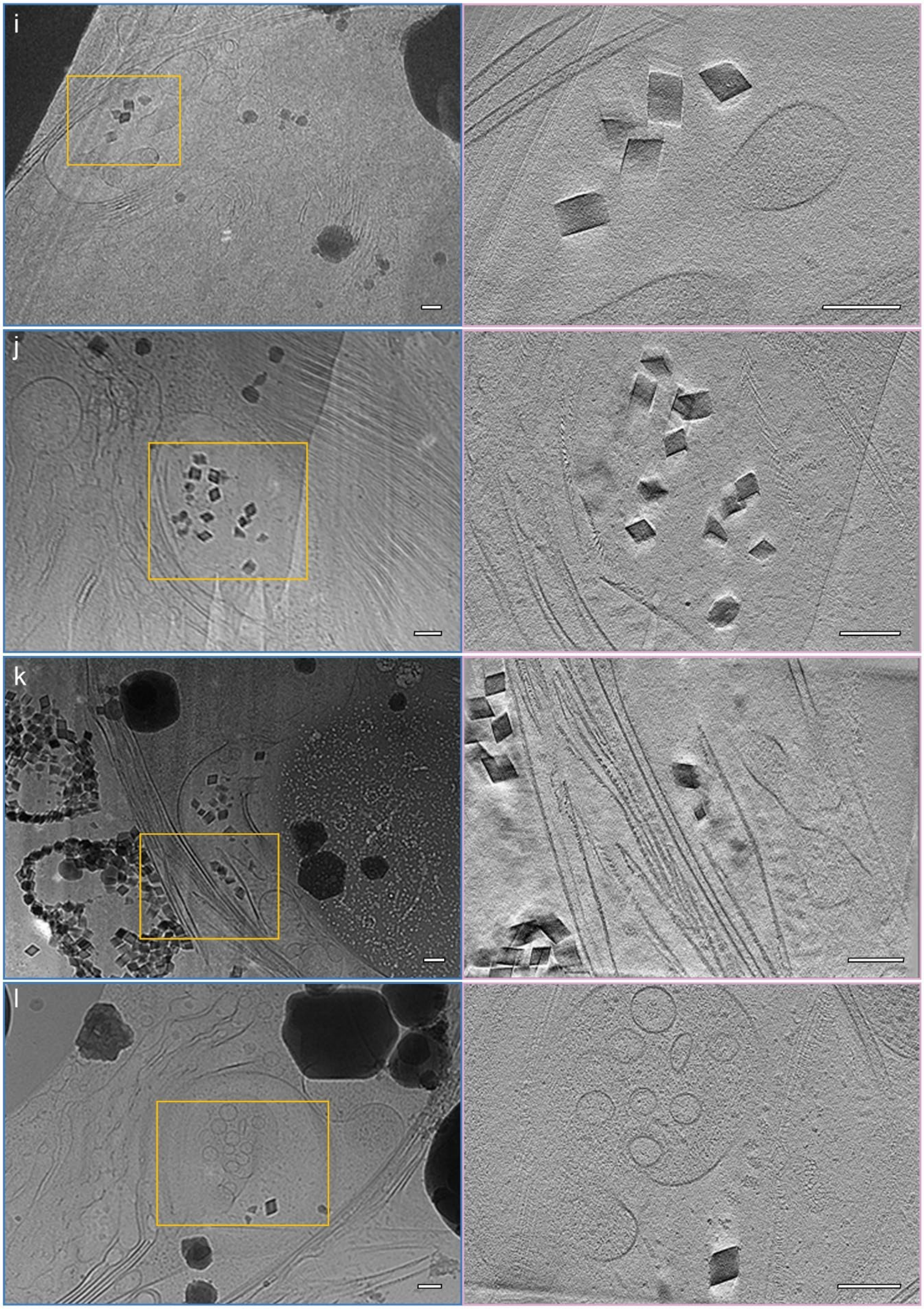

Figure S1 panel 3/4.

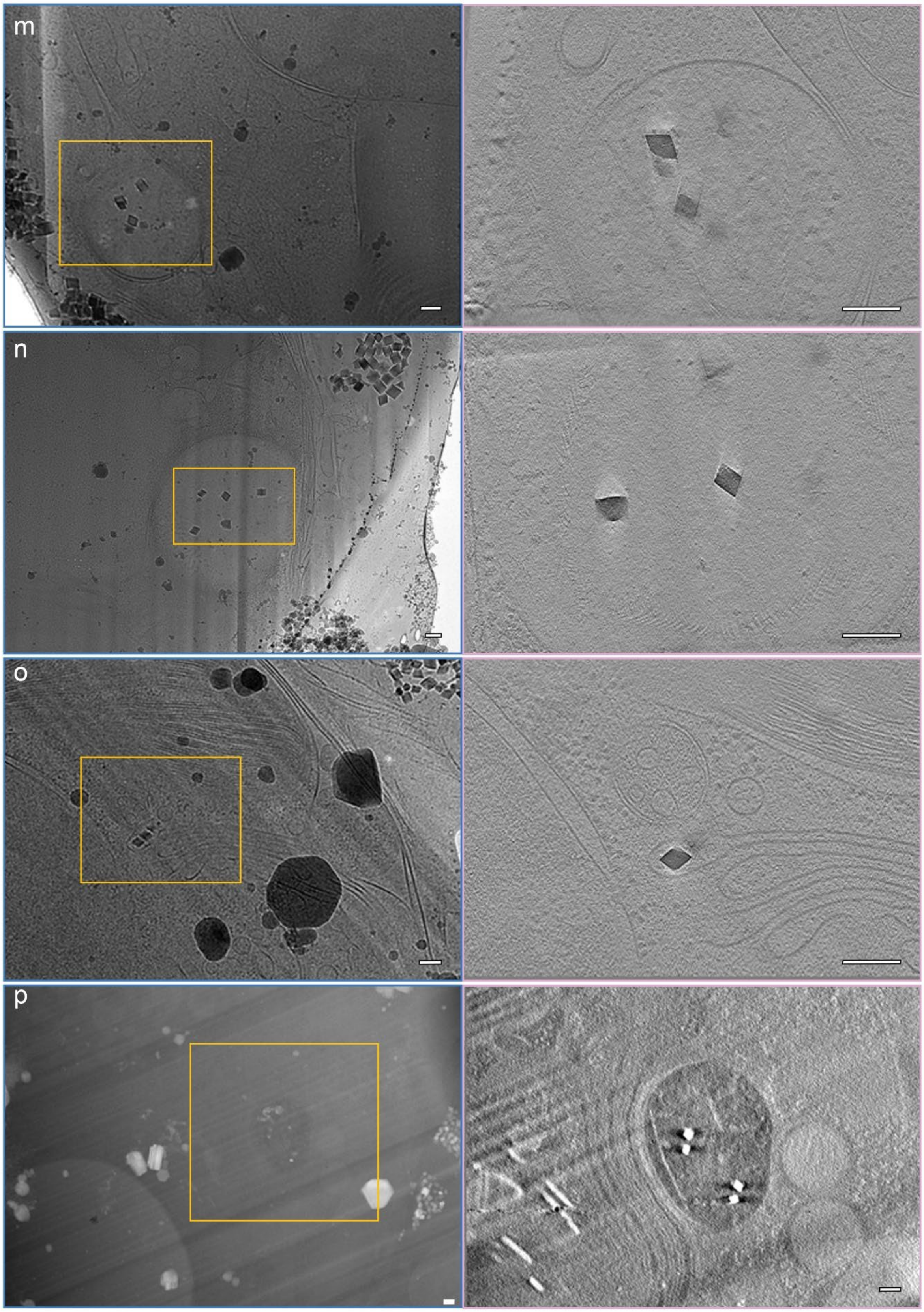

Figure S1 panel 4/4.

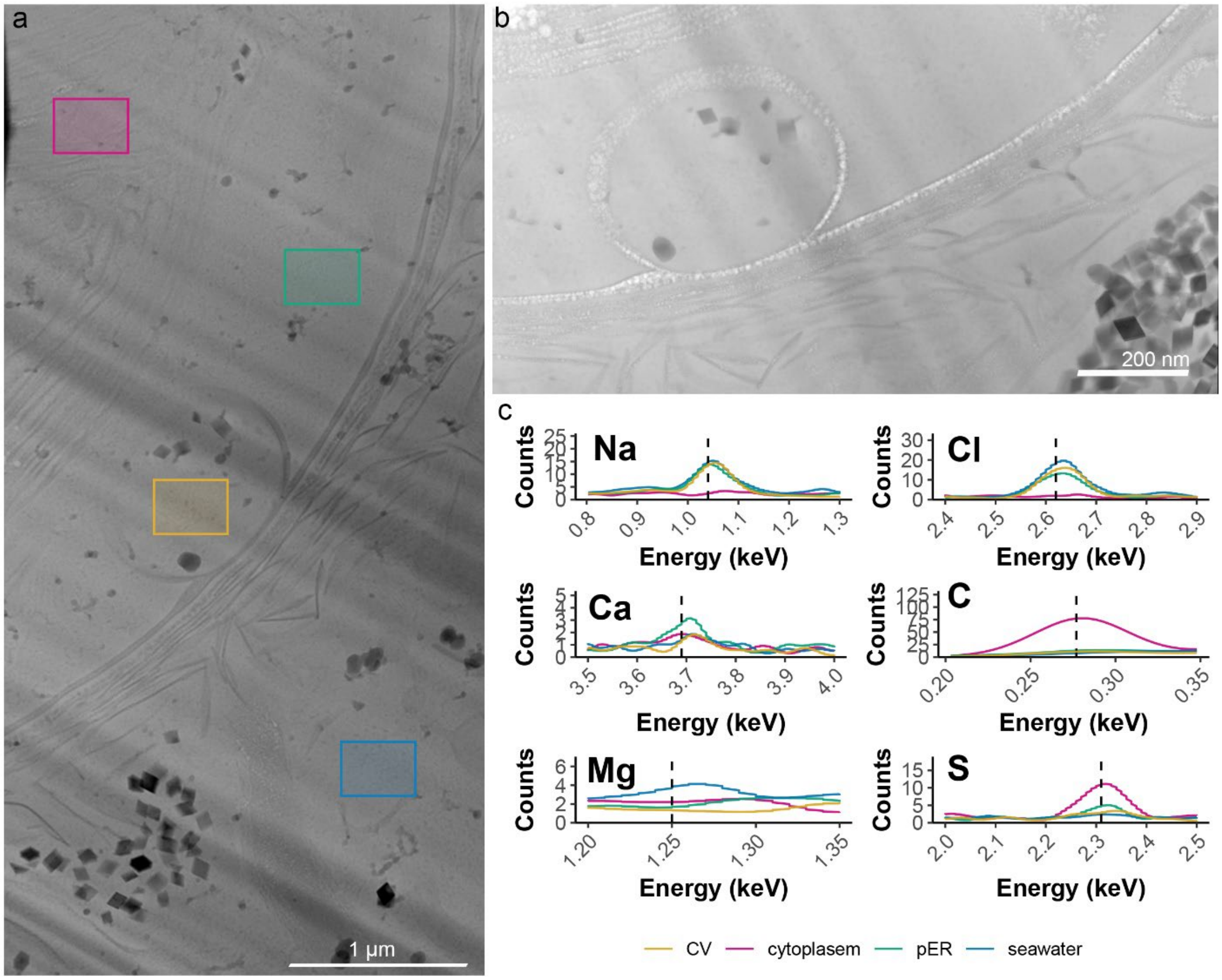

Figure S2. a) Bright-field TEM image of a cellular lamella with regions marked for EDS analysis. Note that the lumen of the CV and pER share a similar gray level. **b**) Higher magnification of CV environment acquired after the STEM-EDS acquisition. Radiation damage is apparent on the CV lipid membrane while the lumen of both the CV and pER seem clear of radiation damage. **c**) EDS spectra of selected elements collected from the color-coded regions shown in a) showing a similar compositional trend to the spectra presented on Fig. 4.
